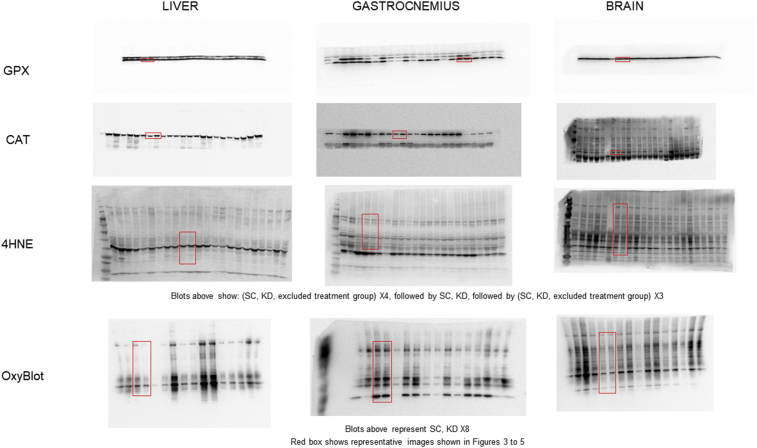# Ketogenic diet increases mitochondria volume in the liver and skeletal muscle without altering oxidative stress markers in rats

**DOI:** 10.1016/j.heliyon.2018.e00975

**Published:** 2018-11-24

**Authors:** Hailey A. Parry, Wesley C. Kephart, Petey W. Mumford, Matthew A. Romero, C. Brooks Mobley, Yufeng Zhang, Michael D. Roberts, Andreas N. Kavazis

**Affiliations:** School of Kinesiology, Auburn University, Auburn, AL, USA

**Keywords:** Metabolism, Nutrition, Physiology

## Abstract

Ketogenic diets (KD) consist of high fat, moderate protein and low carbohydrates. Studies have suggested that KD may influence oxidative stress by affecting mitochondrial quantity and/or quality, and perhaps lengthen lifespan. Therefore, we determined the effects of KD on multi-organ mitochondria volume and oxidative stress markers in rats. Ten month-old male Fisher 344 rats (n = 8 per group) were provided with one of two isocaloric diets: standard chow (SC) or KD. Rats were euthanized if: a) vitality scores exceeded a score of 16, b) rapid weight loss, or c) veterinarian deemed euthanasia necessary. The median lifespan of rats was higher in KD (762 days) compared to SC (624 days). Citrate synthase activity (i.e. estimate of mitochondria volume) was higher in the liver (p = 0.034) and gastrocnemius (p = 0.041) of KD compared to SC. Liver superoxide dismutase 1 and catalase antioxidant protein levels were higher in KD, albeit not significant (p = 0.094 and p = 0.062, respectively). No significant differences in protein levels of other antioxidants or markers of lipid and protein oxidative damage were observed in either the gastrocnemius, liver, or brain. In summary, KD increased mitochondria volume in liver and gastrocnemius and median lifespan in rats. Additionally, our data show that the increase in mitochondrial volume occurred without changes in oxidative damage or antioxidant protein levels in the gastrocnemius, liver, or brain.

## 1 Introduction

Ketogenic diets (KD) are comprised of high fat, adequate protein, and low carbohydrates [1, 2, 3] and have been shown to be effective in treating certain neurological disorders [4, 5, 6]. In addition, there is strong evidence to suggest that a KD is effective for weight-loss in humans [7, 8, 9, 10]. KD have been used concurrently with exercise to aid weight loss [7, 11, 12], and it has been reported that a KD increased fatty acid mobilization compared to runners who consumed a high-carbohydrate diet [13]. Furthermore, we have previously shown that skeletal muscle mitochondrial coupling of complex II substrates is more efficient in rodents fed a KD [14]. Despite the possible benefits of β-oxidation and metabolic function observed with the consumption of KD [4, 13, 14, 15], the impact of a KD on different tissues in the body has not yet been studied in detail.

In addition to the benefits listed above, previous literature states that KD increased the lifespan of rodents. Specifically, Roberts et al. observed a 13.6% increase in the lifespan of mice fed a KD compared to mice fed a standard chow [16]. The effect on longevity may be due to reduced reactive oxygen species (ROS) emission and oxidative damage [17, 18]. Specifically, ROS emission and accumulation of protein carbonyls have previously been shown to increase with age, and decrease with a calorie restricted diet in mice ranging from 9 to 23 months of age [18]. The aforementioned study also demonstrated state 4 mitochondria respiration was decreased with calorie restriction in the brain, heart, and kidney tissues [18]. Additionally, our lab previously reported an increase in liver total antioxidant capacity and increased antioxidant glutathione peroxidase (GPX) liver protein levels after an 8-month long KD diet in rats [19]. In addition, Newman et al. reported that an intermittent KD (i.e., every other week) reduced midlife mortality in mice [20]. These results suggest that a KD diet could mitigate the molecular changes that come with the aging process, potentially explaining the increase in longevity observed in mice fed a lifelong KD. Interestingly, the aforementioned studies are inconsistent with other researchers who reported that mice fed a KD did not experience increased longevity when compared to mice who were fed a standard chow [21].

The inconsistencies in the literature with regards to a KD influencing lifespan imply a need to investigate the effects of a KD on longevity [16, 20, 21]. Additionally, changes in biomarkers related to liver, skeletal muscle, and brain mitochondrial physiology and oxidative stress following lifelong KD have not been fully elucidated. Therefore, the purpose of this study was to determine whether the KD leads to changes in antioxidant or oxidative damage biomarkers in skeletal muscle, liver, and brain.

## 2 Materials and methods

### 2.1 Animals

All experimental procedures were approved by Auburn University's Institutional Animal Care and Use Committee (IACUC, protocol # 2016-2814). Sixteen male Fisher 344 rats at 10 months of age were purchased (Harlan Laboratories, Indianapolis, IN, USA) and allowed to acclimate in the animal housing facility for 1 week prior to experimentation. During acclimation, animals were provided standard rodent chow (SC; 24% protein, 58% CHO, 18% fat; Teklad Global #2018 Diet, Harlan Laboratories) and water ad libitum in 24 °C and constant 12 h light: 12 h dark cycle.

After acclimation, rats were provided isocaloric amounts of one of two diets for the remaining of the study:

1) Eight animals were provided 20 g/day of the aforementioned SC given during the acclimation phase.
2) Eight animals were provided with 16 g/day of a commercially designed KD (Tekland diet #10787) that was designed to induce nutritional ketosis. Casein protein (Optimum Nutrition Inc., Downers Grove, IL, USA) and cellulose powder (Allergy Research Group, Alameda, CA, USA) were added to better compensate for between group differences in protein and fiber content. The diet specifications (post modifications) were as follows: 4.15 kcal/g, 23% protein, 10% carbohydrate (2.9% fiber w/w), and 67% fat. Medium chain triglycerides, flaxseed oil and canola oil were prominent fat sources in the parent KD. Importantly, we have previously shown that this diet induces ketosis in rats [19].

Rats lived out their natural lifespan and were euthanized if: a) vitality scores (range = 4 (good health) to 20 (poor health)) exceeded a score of 16 per the recommendations of Phillips et al. [22], b) rapid weight loss accompanied by changes in food and water consumption, or c) the rat suffered from a condition to which a university veterinarian deemed euthanasia necessary for humane purposes. Rats were euthanized under CO_2_ gas in a 2 L induction chamber (VetEquip, Inc., Pleasanton, CA, USA) according to the American Veterinary Medical Association Guidelines for the Euthanasia of Animals. The gastrocnemius, liver, brain, and adipose tissue pads were dissected out and weighed. The gastrocnemius, liver, and brain were saved and stored at -80 °C and used for subsequent analyses.

### 2.2 Western blotting for oxidative stress measurements

Approximately 60–90 mg of tissue (i.e., gastrocnemius, liver, and brain) was placed in 1x non-denaturing cell lysis buffer (5 mM Tris HCL, 5mM EDTA) and phosphatase inhibitors (2.5 mM pyrophosphate, 1 mM β-glycerophoshate, 1 mM sodium orthovanadate). Samples were then homogenized via micropestle manipulation, and insoluble proteins from homogenates were removed with centrifugation at 1500×g for 10 min. Homogenates were then stored at −80 °C. Protein determination on cell lysis homogenates was performed via Bradford Assay [23]. Homogenates were prepared for Western blotting using 4x Laemmli buffer at 1.5 μg/μL. Subsequently, 15 μl of prepped sample were separated by polyacrylamide gel electrophoresis. After electrophoresis, the proteins were transferred to polyvinylidene difluoride membranes (Amresco, Solon, OH, USA) for 2 h at 200 mA. Nonspecific sites were blocked for 1 h at room temperature in TBS solution containing 0.05% Tween and 5% nonfat milk. Membranes were then incubated for 1 h with primary antibodies directed against the proteins of interest. The primary antibodies used were superoxide dismutase 1 (SOD1; # GTX100554; GeneTex, Irvine, CA, USA), superoxide dismutase 2 (SOD2; # GTX116093; GeneTex), catalase (CAT; # GTX110704; GeneTex), glutathione peroxidase (GPX; # GTX116040; GeneTex), peroxisome proliferator-activated receptor gamma coactivator 1-alpha (PGC-1α; # GTX37356; GeneTex), and 4-hydroxynonenal-conjugated proteins (4-HNE, # ab46545; Abcam, Cambridge, MA, USA). In addition, protein carbonyls were determined using the Oxyblot kit (EMD Millipore; Belliricia, MA, USA) as outlined by the manufacture instructions and previously reported by our laboratory. Briefly, gastrocnemius, liver, and brain homogenates were derivatized to 2,4-dinitrophenylhydrazone (DNP-hydrazone) by a reaction with 2,4-dinitrophenylhydrazine (DNPH). The DNP-derivatized protein samples were separated by polyacrylamide gel electrophoresis, transferred to polyvinylidene difluoride membranes, and incubated with the primary antibody provided in the kit. Following incubation with primary antibodies, all membranes were washed extensively with TBS–Tween and then incubated with secondary antibodies. Membranes were then developed using an enhanced chemiluminescent reagent (Amersham, Pittsburgh, PA, USA), and band densitometry was performed through the use of a UVP Imager and associated densitometry software (UVP, LLC, Upland, CA, USA). Ponceau staining was used as the normalizing control.

### 2.3 Citrate synthase activity

Gastrocnemius, liver, and brain homogenate citrate synthase activity was measured as a function of the increase in absorbance from 5,5′-dithiobis-2-nitrobenzoic acid reduction [24]. Enzyme activities were normalized to total protein levels.

### 2.4 Statistical analysis

All data are presented as means ± standard deviation. A two-way (diet*time) ANOVA was performed for body mass analysis. An independent samples t-test was performed to compare tissue mass at sacrifice, and for all protein expression analysis. Statistical significance was set at p <0.05.

## 3 Results

### 3.1 End point criteria

Of the SC rats, three were euthanized due to vitality scores exceeding 16 points, one was euthanized due to excessive loss in body mass, and four were euthanized based on veterinarian recommendation. Of the KD rats, five were euthanized due to vitality scores exceeding 16 points and three were euthanized due to excessive loss in body mass. Our data show that KD rats had increased median lifespan compared to SC rats (KD = 762 days; SC = 624 days).

### 3.2 Anthropometric measurements

**Fig. 1:**
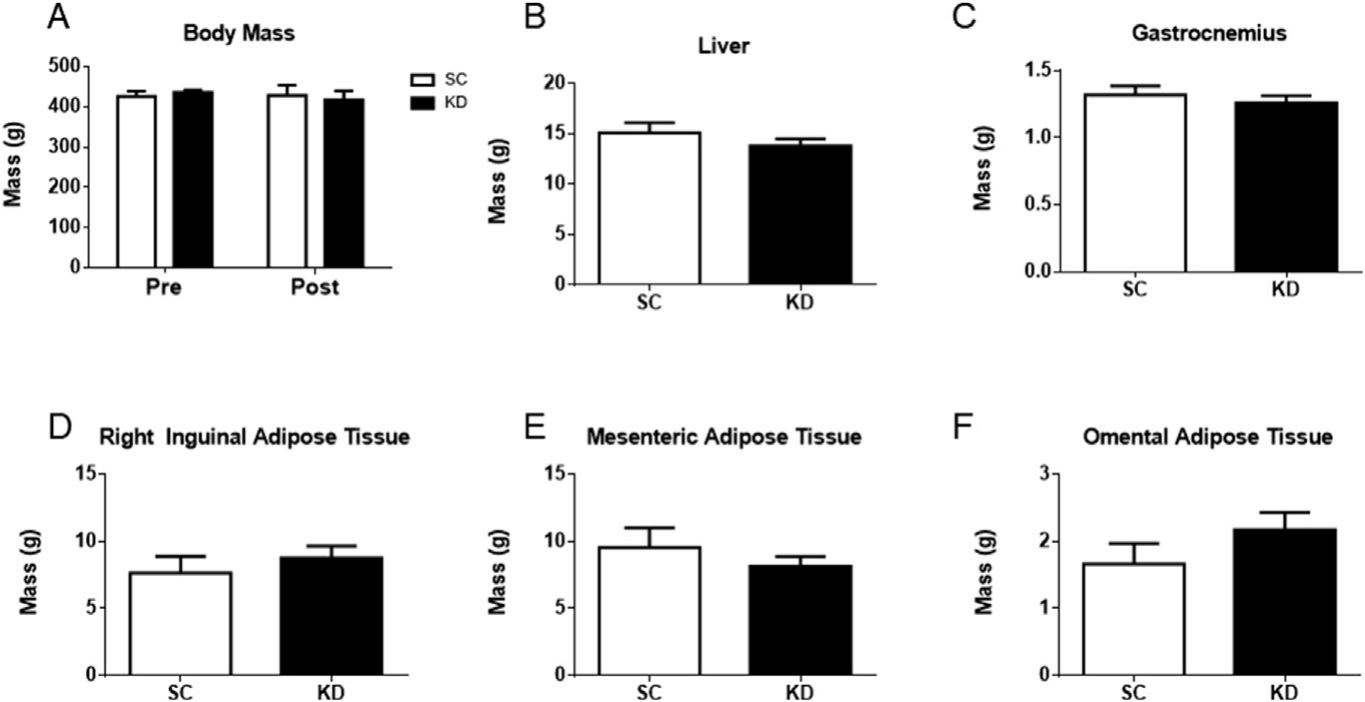
Body and organ masses. n = 8 per group. No significant differences were detected for body mass (A) (p = 0.507), liver (B) (p = 0.314), gastrocnemius (C) (p = 0.509), right inguinal adipose tissue (D) (p = 0.475), mesenteric adipose tissue (E) (p = 0.384), or omental adipose tissue (F) (p = 0.228) between SC and KD rats.

No significant difference was observed in body mass between SC and KD (diet*time: p = 0.507, diet: p = 0.986, time: p = 0.604) (Fig. 1A). Additionally, no significant difference was detected in the liver mass (p = 0.314) (Fig. 1B), gastrocnemius mass (p = 0.509) (Fig. 1C), right inguinal adipose tissue mass (p = 0.475) (Fig. 1D), mesenteric adipose tissue mass (p = 0.384) (Fig. 1E), or omental adipose tissue mass (p = 0.228) (Fig. 1F) between SC and KD.

### 3.3 Citrate synthase activity

**Fig. 2:**
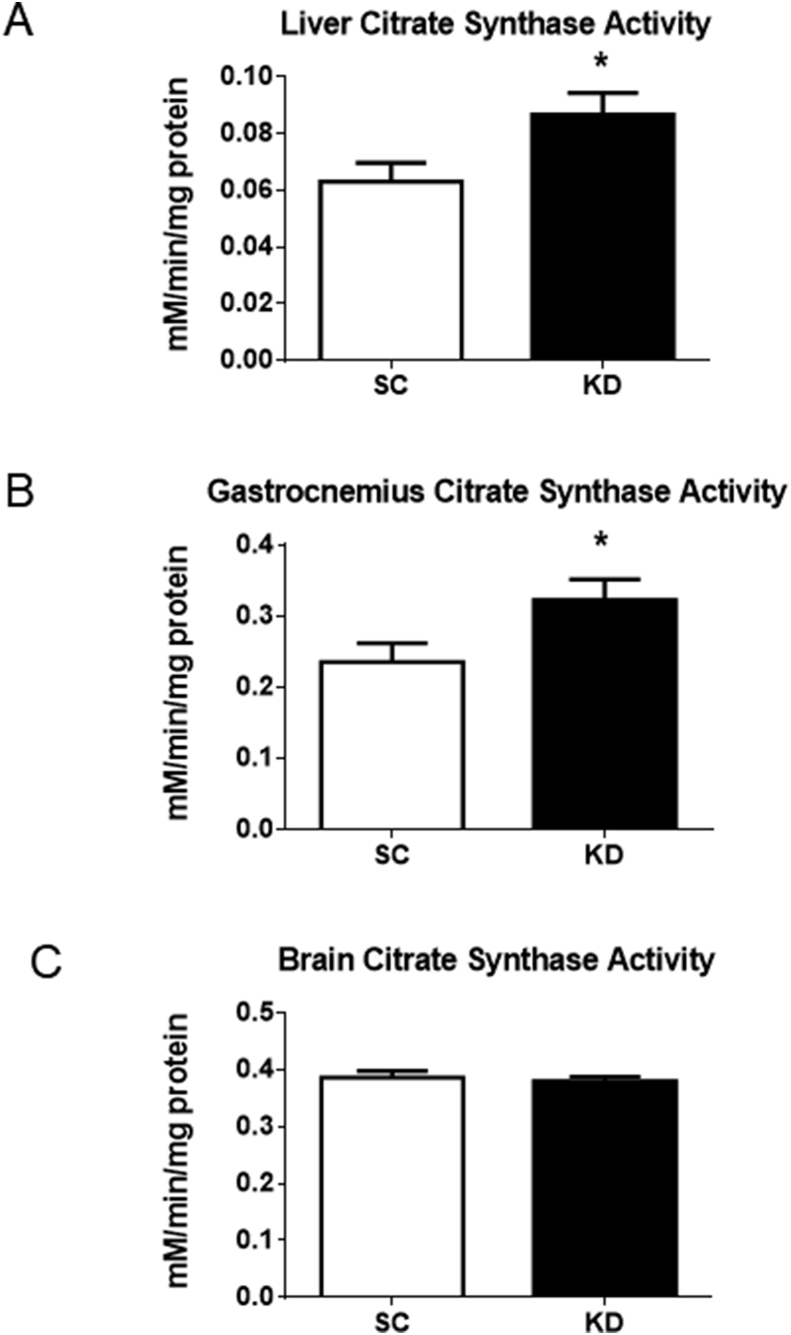
Citrate synthase activity. n = 8 per group. * KD rats had significantly higher liver (A) (p = 0.034) and gastrocnemius (B) (p = 0.041) citrate synthase activity than SC rats. No significant difference was detected for brain (C) citrate synthase activity (p = 0.679).

Citrate synthase activity was measured in the liver, gastrocnemius, and brain (Fig. 2). The KD diet significantly increased citrate synthase activity in the liver and gastrocnemius (p = 0.034 and p = 0.041, respectively; Fig. 2A and B). No differences in citrate synthase activity was observed in the brain (p = 0.679) (Fig. 2C).

### 3.4 Liver antioxidants and markers of oxidative damage

**Fig. 3:**
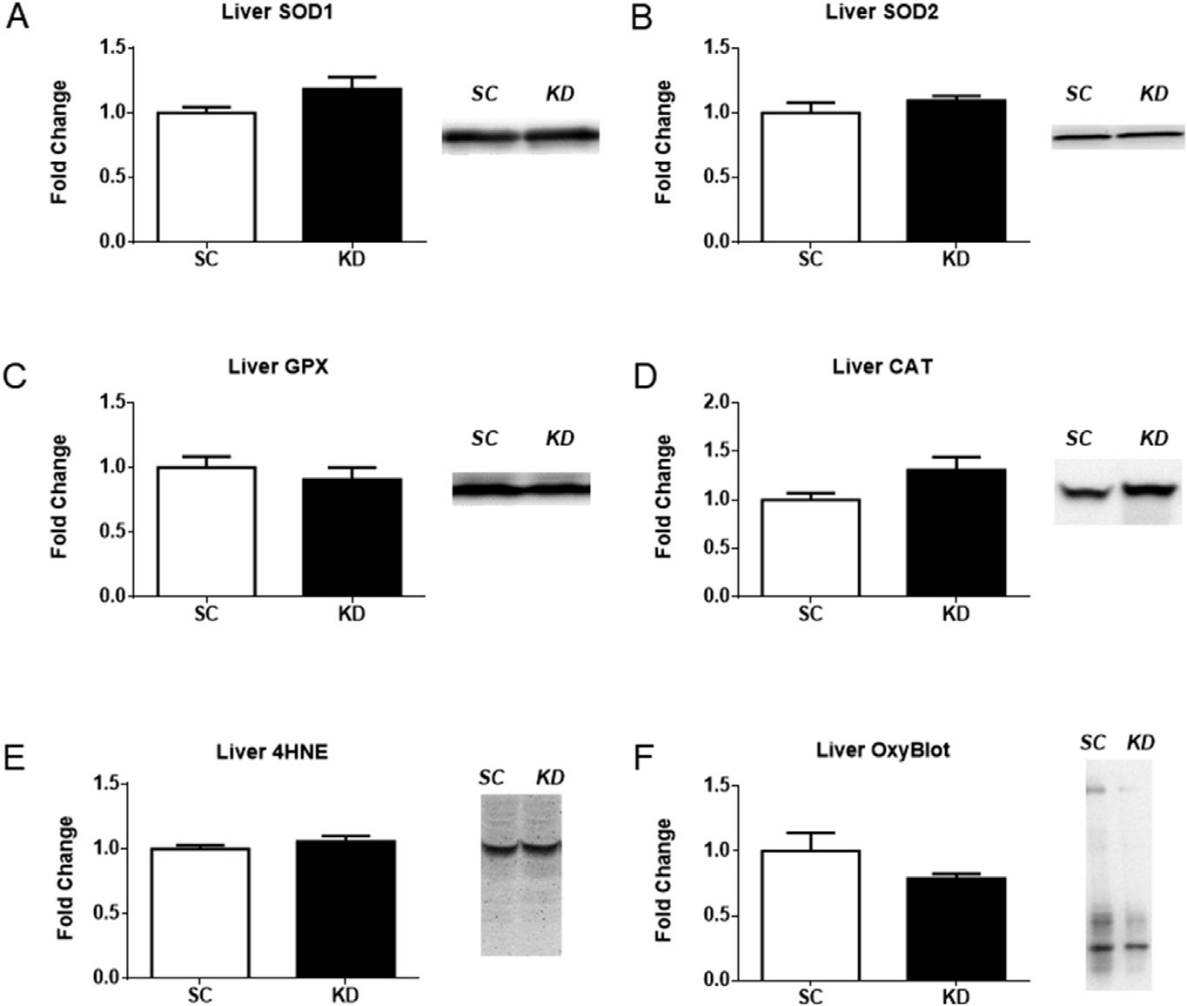
Antioxidant proteins and markers of oxidative damage in liver. n = 8 per group. A trend observed was detected for SOD1 (A) (p = 0.094) and CAT (D) (p = 0.062). No significance was observed for SOD2 (B) (p = 0.315) and GPX (C) (p = 0.473) or the two markers of oxidative damage, 4-HNE (E) (p = 0.276) and OxyBlot (F) (p = 0.197). Representative western blot images are shown to the right of each bar graph. Full images of western blots are presented in supplementary Fig. 1 and 2.

The protein levels of four antioxidants (SOD1, SOD2, GPX, and CAT) and two markers of oxidative damage (4-HNE and OxyBlot) were measured in the liver (Fig. 3). The absolute values of SOD1 (p = 0.094, Fig. 3A) and CAT (p = 0.062, Fig. 3D) protein levels were higher in KD, albeit not significant. Specifically, mice fed the KD showed 18.5% increase in SOD1 and 30.5% increase in CAT. No significance was observed in the antioxidants SOD2 (p = 0.315) (Fig. 3B) and GPX (p = 0.473) (Fig. 3C) or the two markers of oxidative damage, lipid peroxidation (4-HNE, p = 0.276) and protein carbonyls (OxyBlot, p = 0.197) (Fig. 3E and F, respectively).

### 3.5 Gastrocnemius antioxidants and markers of oxidative damage

**Fig. 4:**
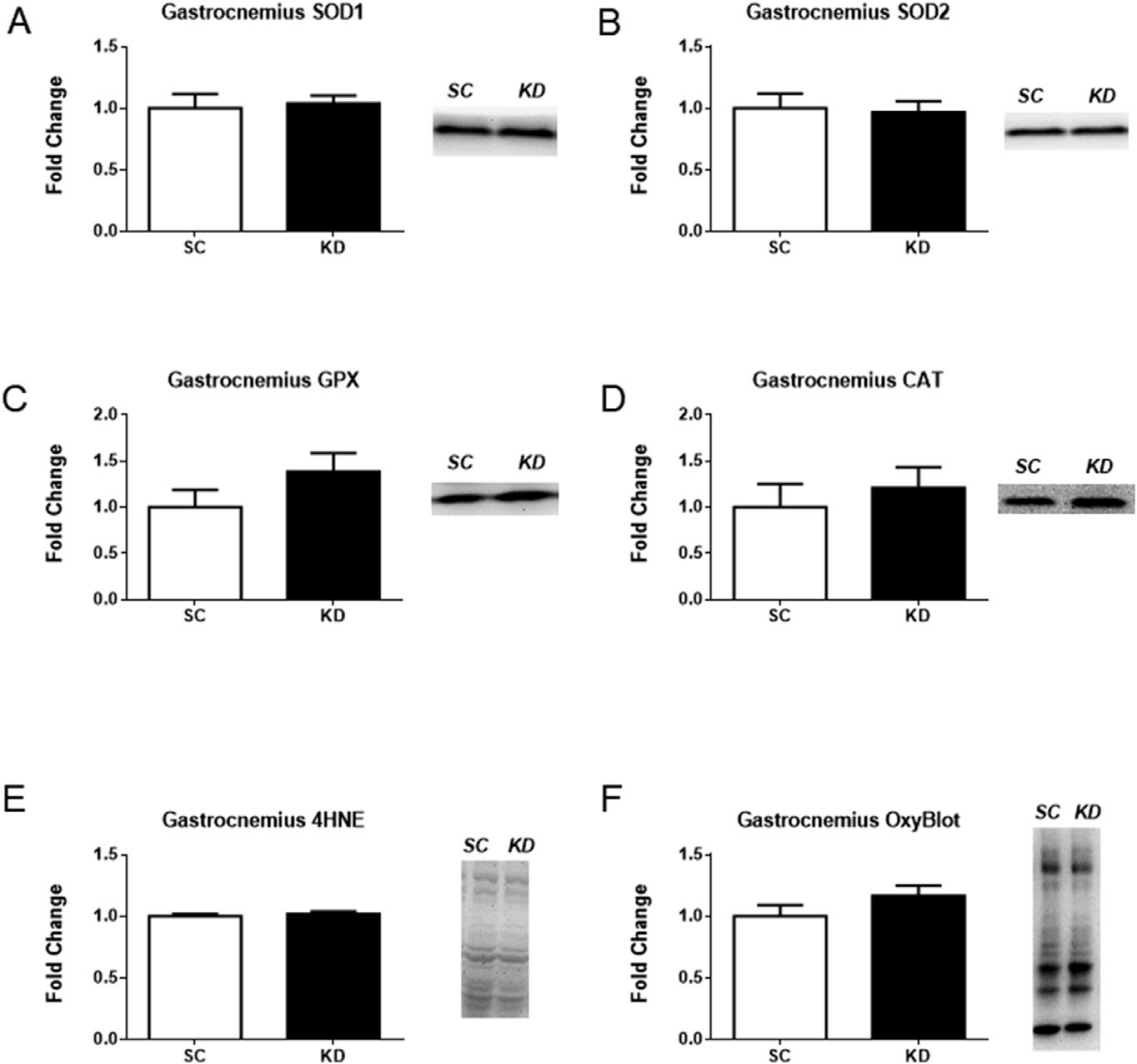
Antioxidant proteins and markers of oxidative damage in the gastrocnemius. n = 8 per group. No significant differences were detected for SOD1 (A) (p = 0.769), SOD2 (B) (p = 0.834), CAT (C) (p = 0.539), and GPX (D) (p = 0.186) or the two markers of oxidative damage, 4-HNE (E) (p = 0.455) and OxyBlot (F) (p = 0.197). Representative western blot images are shown to the right of each bar graph. Full images of western blots are presented in supplementary Fig. 1 and 2.

There were no significant differences in the protein levels of the four antioxidants (SOD1 (p = 0.769), SOD2 (p = 0.834), GPX (p = 0.186), and CAT (p = 0.539)) or the two markers of oxidative damage (4-HNE (p = 0.455) and OxyBlot (p = 0.197)) measured in the gastrocnemius (Fig. 4A–F). Also, PGC-1α protein levels in the gastrocnemius was not different between diets (SC: 1.000 ± 0.097, KD: 1.104 ± 0.085, p = 0.438, units are fold change compared to SC).

### 3.6 Brain antioxidants and markers of oxidative damage

**Fig. 5:**
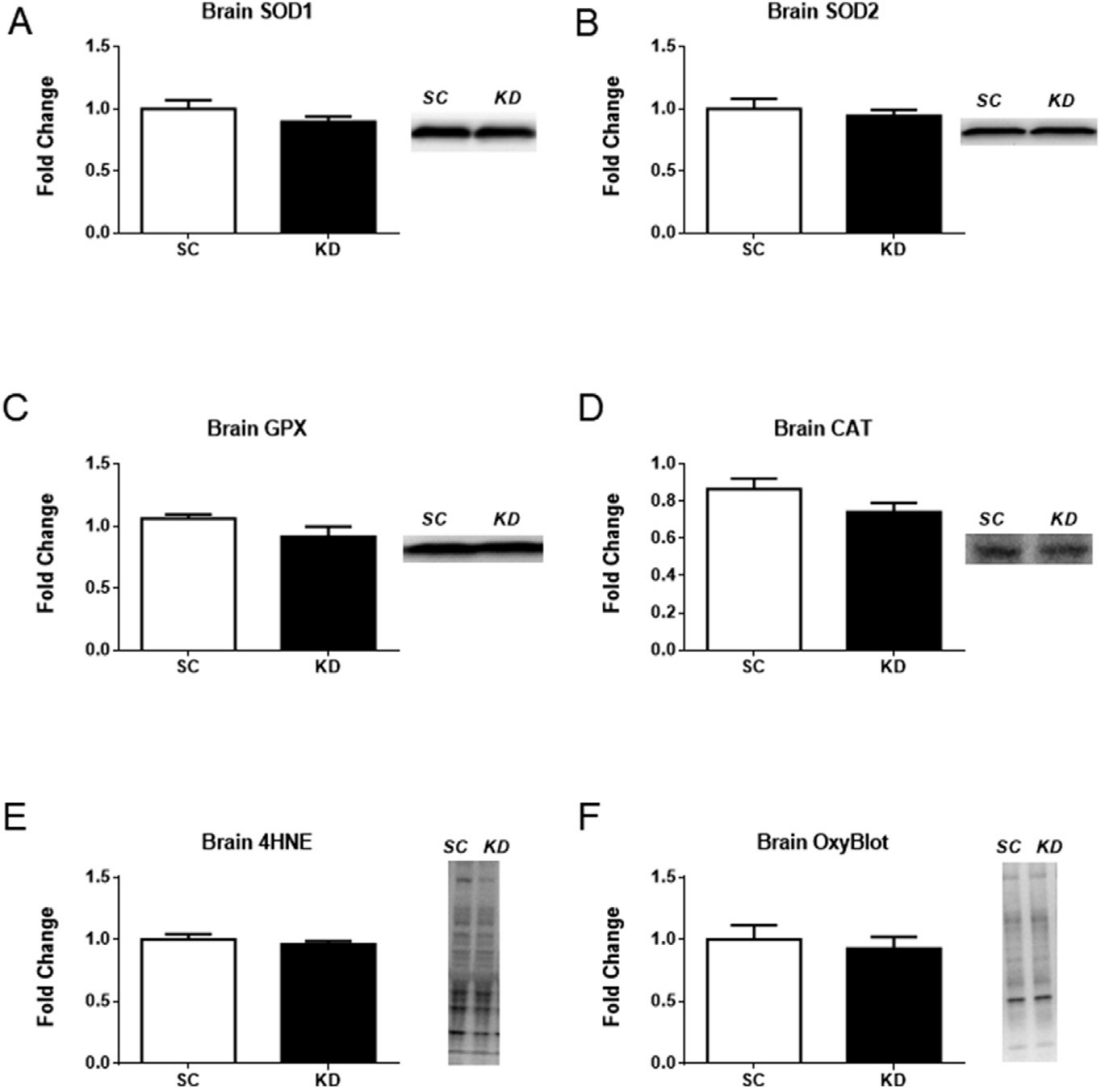
Antioxidant proteins and markers of oxidative damage in brain. n = 8 per group. No significant differences were detected for SOD1 (A) (p = 0.234), SOD2 (B) (p = 0.570), CAT (C) (p = 0.125), and GPX (D) (p = 0.135) or the two markers of oxidative damage, 4-HNE (E) (p = 0.452) and OxyBlot (F) (p = 0.625). Representative western blot images are shown to the right of each bar graph. Full images of western blots are presented in supplementary Fig. 1 and 2.

There were no significant differences in the protein levels of the four antioxidants (SOD1 (p = 0.234), SOD2 (p = 0.570), GPX (p = 0.135), and CAT (p = 0.125)) or the two markers of oxidative damage (4-HNE (p = 0.452) and OxyBlot (p = 0.625)) measured in the brain (Fig. 5A–F).

## 4 Discussion

The benefits of KD for weight loss and therapy for neurological pathologies are well known [4, 25, 26, 27]. Previous studies have investigated the metabolic profile, oxidative stress, and possible longevity benefits of KD, but the results of these studies are equivocal [14, 19, 28, 29, 30]. Therefore, the purpose of this study was to determine whether the KD leads to changes in antioxidant or oxidative damage biomarkers in skeletal muscle, liver, and brain in rats. Results of the current study demonstrate an increase in median lifespan in KD rats with no changes in liver, skeletal muscle or brain oxidative stress markers despite the significant increase in citrate synthase activity in liver and muscle tissues.

Our results are in agreement with the results of Roberts et al. [16] who also observed an increase in longevity in mice fed a lifelong KD. Specifically, researchers observed a 13.6% increase in median lifespan of KD mice compared to mice fed a SC [16]. However, Douris et al. fed mice a KD or SC diet and showed no change in longevity between the diets [21]. Despite the differences in longevity outcomes, both researchers did observe a decrease in lean mass and fat mass and an increase in insulin sensitivity and glucose tolerance [16, 21].

The Free Radical Theory of Aging was first suggested by Denham Harman in 1956 [17, 31]. Specifically, the theory suggests that an increase in metabolism increases oxygen free radicals and other reactive oxygen species (ROS) production, leading to damage which may affect longevity [31]. Production of ROS mainly occurs during oxidation-reduction reactions at complex I and III of the electron transport chain in the mitochondria [32]. Researchers have suggested that if the Free Radical Theory of Aging is correct, then aging may be prevented either by increasing antioxidants, increasing repair/turnover of the proteins damaged by ROS, or decreasing ROS production [17]. For example, researchers have proposed if ROS production increases with age [18], then dietary interventions that would result in a decrease of ROS production could be a way to reverse the effects of aging [17]. Sohel et al. supported this hypothesis when investigating the effects of ROS production, protein carbonyls, and antioxidants in mice when fed a calorie restricted diet [18]. Specifically, the mice fed the calorie restricted diet had reduced ROS production and less protein carbonyls compared to mice fed ad libitum [18]. Our results show that oxidative damage (i.e., 4-hydroxynonenal-conjugated proteins and protein carbonyls) was not significantly different in liver, skeletal muscle, or brain between rats fed a SC or KD. Also, we did not observe any significant differences between groups in protein levels of four antioxidants (SOD1, SOD2, GPX, and CAT) in any of the three tissues studied even though there was an increase in CAT and SOD1 in KD compared to SC, albeit not significant (p = 0.062 and p = 0.094, respectively). These results suggest that the observed increase in the median lifespan of KD rats was not due to changes in the antioxidants and oxidative damage markers measured in this study.

Interestingly we observed that skeletal muscle and liver citrate synthase activity increased in KD-fed rats. This metric was used as an estimation of mitochondrial volume per the findings of Larsen et al. [33] suggesting citrate synthase activity highly correlates with transmission electron micrograph (TEM) images of mitochondrial content (r = 0.84, p <0.001). These results are consistent with past literature which suggest KD feeding can increase mitochondrial volume and function. For instance, a previous study investigated the effects of a Western diet (WD) or a KD for 6 weeks with or without voluntary exercise via a resistance-loaded running wheel in rats [14]. Researchers observed an increase in mitochondrial adaptation in both the WD and KD when rats were exercised. In addition, there was an increase in complex I respiratory control ratio (RCR), albeit not significant (p = 0.07), and a significant increase in complex II RCR in KD rats independent of exercise [14]. Additionally, other studies reported high fat diets increase mitochondrial function in skeletal muscle [34, 35], possibly due to the increased activation of AMPK [36]. It is notable that we recently reported 8 months of KD feeding decreased skeletal muscle citrate synthase activity suggestive of impaired mitochondrial volume [19]. The same study also observed a decrease in complex I RCR in 8-month KD fed rats in skeletal muscle and no change in complex II RCR [19]. These divergent findings may be due to age differences of rats in our previous long-term feeding study. Namely, rats in the current study reached full maturity at 10 months of age prior to the initiation of the feeding intervention, whereas rats in our 8-month feeding study were fed during the growth phase from 4-12 months of age. In lieu of these divergent findings, future investigations should more thoroughly examine the broad-range mitochondrial adaptations that occur with KD feeding, and determine whether the age of rodents upon feeding plays a factor in facilitating differential mitochondrial adaptations.

In summary, KD increased mitochondria volume in liver and gastrocnemius and median lifespan in rats. Additionally, our data show that the increase in mitochondrial volume occurred without changes in oxidative damage or antioxidant protein levels in the gastrocnemius, liver, or brain.

## Declarations

### Author contribution statement

Hailey A. Parry: Performed the experiments, Analyzed and interpreted the data, Wrote the paper.

Wesley C. Kephart: Performed the experiments, Analyzed and interpreted the data.

Petey W. Mumford, Matthew A. Romero, C. Brooks Mobley, Yufeng Zhang: Performed the experiments.

Michael D. Roberts, Andreas N. Kavazis: Conceived and designed the experiments, Contributed reagents, materials, analysis tools or data.

### Funding statement

This work was supported by a contract from Applied Sports Performance Institute to Michael D. Roberts.

### Competing interest statement

The authors declare no conflict of interest.

### Additional information

No additional information is available for this paper.

## Figures and Tables

**Supplementary Figure 1:**
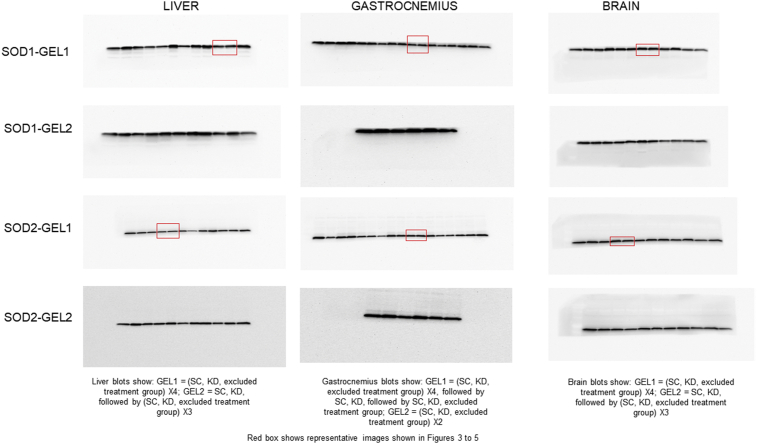


**Supplementary Figure 2:**